# Treatment of Misophonia with Risperidone in a Patient with Autism Spectrum Disorder

**DOI:** 10.1155/2022/3169834

**Published:** 2022-09-30

**Authors:** Eric J. Pan, Jeremy Weleff, Akhil Anand, Brian S. Barnett

**Affiliations:** ^1^Case Western Reserve University School of Medicine, 9501 Euclid Ave, Cleveland, OH, USA 44106; ^2^Cleveland Clinic, Psychiatry and Psychology, 9500 Euclid Ave, Cleveland, OH, USA 44106; ^3^Department of Psychiatry, Yale University School of Medicine, New Haven, Connecticut, USA

## Abstract

We report the case of a 32-year-old male with autism spectrum disorder (ASD) suffering from severe misophonia. After titrating risperidone to 2 mg twice a day, the patient reported a significant reduction in his symptoms and his Amsterdam misophonia scale-revised (AMISOS-R) score dropped by from 31 to 5. Upon discharge, the patient was noted to have decreased irritability and overall improved behavior and effect. This significant symptomatic improvement was likely not explained by inpatient admission alone or other simultaneous pharmacologic treatments, as the effect was seen during an isolated titration of risperidone with other treatments remaining constant. Although, unfortunately, follow-up findings indicated that the treatment was not curative for the patient, risperidone's potential for treating misophonia may warrant systematic investigation.

## 1. Introduction

Autism spectrum disorder (ASD) is a neurodevelopmental disorder characterized by communication and social skills impairment, repetitive and restricted behaviors, and sensory processing disorders (SPD). At age eight, the prevalence of ASD is around 1.5 percent [1]. The prevalence of SPDs in the general children population is approximately 5-15 percent. Patients with ASD will also have a comorbid SPD in 80-90 percent of cases [2–5]. Misophonia, a type of SPD in which specific sounds and sound patterns elicit a strong negative emotional response and autonomic “fight-or-flight” reaction, is seen in a fraction of individuals with ASD [6]. Triggers often include repetitive sounds such as chewing, pen clicking, tapping, loud breathing, or lip-smacking. Patients are often driven to avoid situations where these sounds may arise and use coping mechanisms like headphones or even pillows over their heads in order to placate their autonomic arousal when they are encountered [7]. Misophonia is posited to result from hyperconnectivity between the auditory and limbic systems [8]. The emotion primarily associated with misophonia is anger, as opposed to the fear seen in phonophobia. Along with anger, feelings of irritation, stress, anxiety, aggravation, being trapped, and impatience can also occur [9, 10]. The specific sound cue is believed to activate the right insula, right anterior cingulate cortex, and the right superior temporal cortex, resulting in the intense negative reaction experienced by the individual [11]. While gaining more recognition, many questions remain about the prevalence, phenomenology, and treatment of this condition. Evidence for effective treatment of misophonia is scarce. To date, no controlled trials appear to have been conducted, resulting in most data on misophonia treatment being anecdotal. Case studies have explored using cognitive behavioral therapy (CBT), exposure and response prevention therapy, mindfulness, and selective serotonin reuptake inhibitors (SSRIs) in treating misophonia with mixed results [12–14]. A recently published randomized controlled trial of CBT has shown it to be effective for patients with misophonia [15]. However, we are unaware of any randomized controlled trial of pharmacological agents for this condition.

Hyperacusis, another form of auditory SPD, shares some similarities with misophonia. However, while everyday sounds can be extremely painful and loud in hyperacusis; in patients with misophonia, it is a specific sound that produces an aversive reaction [16]. While case reports support using risperidone as a potential treatment option for hyperacusis in patients with autism, we are unaware of published reports on using risperidone to treat misophonia in this patient population [17, 18]. Here, we discuss the case of a 32-year-old male with ASD whose misophonia responded dramatically to risperidone.

## 2. Case Presentation

A 32-year-old male with ASD, intellectual disability, generalized anxiety disorder, and schizoaffective disorder was brought to the emergency department by the family for concerns of acute-onset paranoia and delusions in the setting of an olanzapine taper initiated by his outpatient psychiatrist due to lack of efficacy for psychosis and experienced weight gain. The patient was admitted to our psychiatric unit after reporting paranoia over the smoke detectors in his home and was worried his neighbors were spying on him and having a staring episode that raised concern for seizures. At time of admission, his general anxiety disorder-7 (GAD-7) score was 20 and patient health questionnaire-9 (PHQ-9) score was 14 [19, 20]. On admission, the patient was continued on olanzapine 20 mg at bedtime and started on risperidone 1 mg, which was increased to 1 mg twice a day the following day with a plan to cross-taper. His home divalproex delayed release 750 mg twice a day, pantoprazole delayed release 40 mg daily, lisinopril-hydrochlorothiazide 10-12.5 mg daily, pravastatin 20 mg daily, topiramate 50 mg twice a day, and venlafaxine extended release 150 mg daily were continued. Gabapentin 300 mg three times daily was added for chronic back pain on hospital day 1.

In addition to psychotic symptoms, the patient endorsed having misophonia. He described developing a headache and a negative emotional reaction to various sounds, including the sounds of people eating, talking, snoring, fireworks, and cell phones ringing. Before admission, the patient kept his phone on vibrate at all times for fear of the sound of the ringing. The patient also described multiple instances where he would leave the area, put a pillow over his head, or have a “big blowup” fight with the individuals making the noise where he would scream and become physically aggressive. He also actively avoided a relative who he knew was “a loud eater with breathing issues”, which made him feel guilty, and he often asked his parents to leave the room due to the sounds they made while eating. During admission, the patient initially was noted to be very uncomfortable when in environments with many people talking or eating, such as during group or during meals. In quiet environments, he was observed to be calmer and not in distress. The patient's misophonia was evaluated using the 10-item Amsterdam misophonia scale-revised (AMISOS-R), consisting of 10 items, with a total score ranging from 0-40 (I. [21]). The scale has not been fully validated, but preliminary analysis has demonstrated good reliability. Higher scores indicate higher misophonia severity; our patient scored 31, indicating severe to extreme misophonia [15].

On the psychiatric unit, the patient continued to report paranoia towards others, including staff members. He had a behavioral outburst and refused to speak until the door was closed because he feared others were listening. He also reported experiencing audio and visual hallucinations. After seven days on the psychiatric unit, the patient was transferred to the medical unit for a neurological workup of a suspected seizure on the unit. Neurological workup, including MRI and EEG, were negative, indicating no epileptic waveforms or other findings indicative of seizures. Psychogenic nonepileptic seizures could not be ruled out. The patient was fully tapered off his olanzapine on hospital day seven, after being transferred to the medical service; and the following day, his risperidone was increased to 2 mg twice a day. He tolerated this dosage increase and as detailed below and responded well to it. The patient was then retransferred to inpatient psychiatry on hospital day nine. No further seizure-like activity was noted. On hospital day 12, the patient was discharged and was noted to be future-oriented, denying paranoia, hallucinations, suicidal ideation, and homicidal ideation.

The effect of increasing risperidone to 2 mg twice a day on the patient's misophonia was sudden and dramatic. He was engaged and pleasant for the remainder of his hospital stay, with no further behavioral outbursts, and he attended all groups. His score on the AMISOS-R dropped to five, indicative that the patient's misophonia had become subclinical [15]. On the day of discharge, the patient reported that he was feeling less irritable, more talkative overall, and was no longer bothered by the sounds of people eating or talking, and that those formerly distress-inducing sounds instead “rolled over him.” The patient noted that he could now easily tolerate his roommate's snoring, which he was not able to do before his hospitalization. At the time of discharge, the patient wondered aloud if he would now be able to turn on the ringer on his cell phone and tolerate a family member who was a loud eater.

One month after discharge from this hospitalization, the patient's mother reported that the patient's misophonia remained improved. However, he continued to experience some irritability and sensitivity to noises when the family ate meals together. The patient remains on risperidone, with the dosage increased to 2 mg daily and 3 mg at bedtime following another hospitalization for agitation unrelated to misophonia approximately 2 months after his hospitalization at our facility.

## 3. Discussion

We are unaware of any cases in the literature detailing antipsychotic use to treat misophonia in adult patients with ASD. In this case, the finding of improved misophonia after treatment with risperidone was unexpected, since risperidone was being used to target psychosis. Given the report of the patient still having some symptoms of misophonia once discharged home, risperidone appears to have been helpful for his misophonia, but not curative. The switch from olanzapine to risperidone was the medication change that most likely explained our patient's misophonia improvement. While the patient was started on both gabapentin (for back pain) and risperidone at the start of his hospitalization, it was on hospital day eight, the day after risperidone was increased to 2 mg twice a day, when he noticed dramatic improvement in his misophonia. No change in gabapentin dosage occurred during the admission. We found no reports of gabapentin treating misophonia, though we did locate a report of it treating hyperacusis [22]. Little is known about the underlying biology of misophonia and the role of individual neurotransmitters in this condition. How risperidone may have treated our patient's misophonia, while olanzapine was not able to, is not understood. Both drugs antagonize D2 dopaminergic receptors, but risperidone has a stronger affinity for these receptors. In addition, both drugs bind differentially to a variety of other receptors including, 5-hydroxytryptamine 2 (5-HT2), histamine H1, *α*1-adrenergic, and D1dopamine receptors [23]. More research remain necessary to establish the efficacy of misophonia treatment with risperidone and to elucidate the mechanisms by which risperidone might help treat this potentially debilitating and often misunderstood condition.
